# Long‐Term Adherence and Drug Utilisation Patterns Among New Users of Anti‐Hyperlipidemic Monotherapy: Development of a Risk Prediction Model

**DOI:** 10.1111/jep.70380

**Published:** 2026-02-18

**Authors:** Xuechun Li, Eelko Hak, Jens H. J. Bos, Catharina C. M. Schuiling‐Veninga, Sumaira Mubarik

**Affiliations:** ^1^ Unit PharmacoTherapy, ‐Epidemiology and ‐Economics, Groningen Research Institute of Pharmacy University of Groningen Groningen the Netherlands

**Keywords:** adherence, drug pattern, logistic regression, risk factors, statin

## Abstract

**Rationale:**

Real‐world long‐term adherence and drug patterns are essential in hyperlipidemia management. However, the evidence was unclear.

**Aims and Objectives:**

This study aimed to assess long‐term adherence, drug usage patterns of anti‐hyperlipidemic monotherapy, associated risk factors, and develop risk prediction models.

**Methods:**

We performed a retrospective inception cohort study utilising data from the University of Groningen's IADB.nl dispensing database. The study included new users of anti‐hyperlipidemic monotherapies—simvastatin, pravastatin, fluvastatin, atorvastatin, rosuvastatin, and fibrates, with approximately a follow‐up to 10 years. Using logistic regression, we developed risk prediction models for adherence and drug utilisation patterns, including discontinuation, continuation, switching, and add‐on therapies.

**Results:**

Simvastatin users demonstrated high prevalence of high adherence throughout the study (83.6%–90.2%) and had the highest continuation rate (39.2%) with minimal switching or add‐on therapy use. Individuals aged 40 and older exhibited better adherence and higher continuation rates. Male patients had lower adherence but higher continuation rates. High adherence was associated with both high continuation and increased switching or add‐on therapy use. Patients using diabetes medications had better adherence, higher continuation, and lower switching and add‐on rates, whereas those on antiparkinson drugs had lower continuation rates. Recent initiators showed better continuation and lower switching or add‐on rates.

**Conclusions:**

Simvastatin users demonstrated higher adherence and continuation rates compared to other anti‐hyperlipidemic monotherapy users. Factors including older age, female sex, and diabetes medications use were associated with improved adherence. Sensitivity analyses using equivalent dosing regimens yielded consistent findings. These insights into adherence and drug patterns are critical for informing personalised strategies to optimise cardiovascular disease prevention.

**Abbreviations**
AUC: area under the curve
CI: confidence interval
COPD: chronic obstructive pulmonary disease
CVD: cardiovascular diseases
EQD: equivalent doses
HR: hazard ratio
IPW: inverse probability weighting
OR: odds ratio
RA: rheumatoid arthritis
SD: standard deviations

## 1 Introduction

Hyperlipidemia has been confirmed as a risk factor for cardiovascular disease (CVD) [1]. In current daily clinical practice or guidelines, the advice is to prescribe moderate‐ or high‐intensity statins monotherapy as the initial therapy when patients has documented atherosclerotic CVD or high LDL‐cholesterol levels [2, 3]. Despite an increasing number of studies on the effects of combination lipid‐lowering therapy, there is limited evidence to demonstrate its advantage over statin monotherapy [4].

The adherence to anti‐hyperlipidemic medication is crucial in ensuring the efficacy of monotherapy treatment. Non‐adherence to statin treatment can significantly elevate the susceptibility to CVD [5]. Similarly, improving adherence is associated with a lower risk of stroke [6]. Therefore, improving adherence to anti‐hyperlipidemic monotherapy ensures similar effectiveness of monotherapy compared with combination or fixed‐dose combination therapy.

Since lipid‐lowering medications can be used over a long period of time, understanding long‐term patterns of discontinuation, switch and add‐on therapy provides valuable insights for possible prognosis and the alignment with drug guidelines.

Various common risk factors have been confirmed to influence adherence, including traditional risk factors as age, sex and co‐morbidities, for example, having diabetes [7]. Side effects of statins, complexity treatment, medication costs, and psychosocial factors may also play a role [8]. For drug patterns, intolerable side effects (statin‐associated muscle symptoms) may lead to switching or temporary discontinuation [9] whereas for add on treatment, incomplete efficacy of original statin may lead to add on therapy (Ezetimibe) [10].

We aimed to analyze adherence and drug usage patterns among new users of anti‐hyperlipidemic monotherapy aged 18 years and older in the Netherlands, while exploring associations with key risk factors. These findings provide insights into adherence trends, responses to guideline changes, and individual variations in prescribing behaviour.

## 2 Methods

### 2.1 Study Population and Study Design

Data on new users of anti‐hyperlipidemic monotherapies were extracted from the extensively utilised University of Groningen IADB.nl pharmacy dispensing database, covering the period from 1 January 1996 to 31 December 2020. For this retrospective inception cohort study, the inclusion and exclusion criteria aligned with those used in a prior effectiveness study conducted by our research group [11]. The date of the first anti‐hyperlipidemic drug monotherapy was the start time of the follow up. New users of selected anti‐hyperlipidemic drug monotherapy older than 18 years at the start time were entered in the cohort. All eligible patients had at least 2 years of records before the start time and 1 year of data records thereafter. Patients who had records of anti‐hypertensive monotherapies or fixed‐dose combinations of anti‐hypertensive/anti‐hyperlipidemic drug in the year following the start time were excluded. According to our group's earlier study, patients who received acute cardiac drug therapy or possible chronic drug therapy within the 2 years before or 90 days after the start time were also excluded from the analysis. Additionally, the clinical characteristics of patients varied across the different monotherapies [11].

After approximately a 10‐year follow‐up period (180 days ≈ 0.5 year; 360 days ≈ 1 year; 3600–3780 days ≈ 10 years), the study concluded on 31 December 2020. The participants were then categorised into groups based on their total follow‐up duration. The follow‐up period for each participant ended upon the earliest occurrence of one of the following: the study's end date, a cardiovascular event, the date of the final treatment prescription, or the initiation of a treatment change or addition by the patient.

### 2.2 Anti‐Hyperlipidemic Monotherapy as the Exposure

Based on cardiovascular risk management guidelines in the Netherlands [12], simvastatin, atorvastatin and rosuvastatin are preferred over pravastatin and fluvastatin. Pravastatin and fluvastatin are recommended only in specific situations: if the use of atorvastatin, rosuvastatin and simvastatin is not eligible due to side effects or the risk of rhabdomyolysis in interaction with other agents or if other statins are intolerant and have a weaker lipid‐lowering effect. Therefore, study treatment contained statins (C10AA) and fibrates (C10AB) based on this guideline. At least three prescriptions of the same class in the first year since index date were required. The statins were then divided into 5 different compounds, including simvastatin (C10AA01), pravastatin (C10AA03), fluvastatin (C10AA04), atorvastatin (C10AA05) and rosuvastatin (C10AA07).

### 2.3 Adherence and Drug Patterns as Outcome

Outcomes were all defined in patients with specific time risk window but before the maximum total 10‐year follow‐up time or 2020‐12‐31 (see Table 1). The details of the drug patterns were clarified in Table 1 and our previous paper [11].

**Table 1:** The definition and time risk window of outcomes.

Outcomes	Definition	Patients in time risk window
Adherence		
Average adherence	Continuous adherence was defined as the number of covered days within each year (numerator) divided by a fixed denominator of 360 days (because we regarded 180 days as half year, 360 days can be considered as 1 year). Average adherence was then summarised across patients using mean ± standard deviation (SD).	Patients on original antihyperlipidemic monotherapy exceeding 1 year, 2 years …until 10 years.
Binary adherence	High (adherence ≥ 0.8) and low (adherence < 0.8) [13].	Patients on original antihyperlipidemic monotherapy exceeding 1 year.
Prevalence of high adherence	The proportion of high adherence (patients with adherence ≥ 0.8/all the patients).	Patients on original antihyperlipidemic monotherapy exceeding 1 year, 2 years …until 10 years.
3‐year binary adherence	The numerator was the number of covered days during follow up time, while the denominator was the total follow up days, then divided into binary adherence.	Selecting patients on original antihyperlipidemic monotherapy ≤ 3 years in patients with all drug records exceed 3 years.
Drug patterns		
Continuation	Keep using the baseline monotherapy from start time to the end of follow up time without any change.	Patients on original antihyperlipidemic monotherapy exceeding 1 year.
Discontinuation	Stop using the initial antihyperlipidemic monotherapies for more than 180 days.	Patients on original antihyperlipidemic monotherapy exceeding 1 year.
Switch	Changing the initial monotherapy to either a different monotherapy or a fixed‐dose combination within 180 days after discontinuation of the initial treatment.	Patients on original antihyperlipidemic monotherapy exceeding 1 year.
Add_on	Addition of an initial lipid‐lowering drug monotherapy or a fixed‐dose combination before discontinuation.	Patients on original antihyperlipidemic monotherapy exceeding 1 year.

### 2.4 Potential Risk Factors

At the time of initiation, age groups were categorised as follows: 18–39, 40–69, and ≥ 70 years, because the Netherlands CVD risk management guidelines usually consider individuals < 40 and > 70 years as groups in which age may influence CVD risk management [12]. Sex was classified as male or female. Baseline drug‐treated comorbidities were defined by having at least one prescription for the following drugs within the first 180 days from the start time for diabetes, rheumatoid arthritis (RA), or asthma/chronic obstructive pulmonary disease (COPD). Same measurement for the use of antiepileptic drugs, antiparkinson drugs, psycholeptic drugs, psychoanaleptic drugs, addictive disorders drugs, and antineoplastic drugs. Additionally, the calendar year of the first prescription of treatment was categorised as 1996–2000, 2000–2010, or 2010–2020 which was the most recent period, reflecting the evolution of the guidelines and adherence was classified as high (adherence ≥ 0.8) or low (adherence < 0.8) [13].

### 2.5 Statistical Analysis

Average adherence were summarised using mean ± standard deviation (SD), and categorical variables were presented as proportions with percentages. A significance level of *p* < 0.05 indicated statistical significance.

We applied logistic regression using the basic R studio package “stats” to build risk prediction models for binary adherence and drug patterns. Our analysis included treatment, age, sex, baseline drug‐treated comorbidities, as well as calendar year and/or adherence levels. To ensure model robustness, we split the dataset into training and test sets, maintaining a 7:3 ratio [14]. The training set was used to construct the model, while the test set was used to assess the model's predictive performance against actual outcomes. Evaluation metrics included sensitivity, specificity, the area under the curve (AUC), and the Hosmer‐Lemeshow goodness of fit (if *p*‐value greater than 0.05, the model has a good fit).

The outcome 3‐year binary adherence in the following analysis is the level of adherence to anti‐hyperlipidemic drug monotherapy over the 3‐year follow‐up, categorised as either “low adherence” or “high adherence.” A Kaplan‐Meier curve was used to illustrate the probability of remaining in the low adherence group over time. Additionally, inverse probability weighting (IPW) adjusted‐Cox regression analysis, with and without time‐dependent effects, were performed to compare the relative risk (hazard ratio) of transitioning to high adherence among patients on different anti‐hyperlipidemic monotherapy treatments. IPW was applied to balance baseline confounding factors across different treatment groups, thereby reduce bias [15]. All preprocessing and analysis of data were in Rstudio version 4.3.2.

### 2.6 Sensitivity Analysis

We applied the equivalent doses (EQD) scheme to describe the drug switch and drug add‐on. The utilisation of EQD ensured uniformity in LDL cholesterol reduction across various anti‐hyperlipidemic medications [16, 17]. Following the categorisation by Steenhuis et al., [16] dividing EQD into three tiers (reduction in LDL cholesterol: less than 30%, 30%–45% and over 45%), in this case, we categorised patients into comparable levels: low, medium, and high based on baseline doses and their doses of switched or added prescription.

To assess the robustness of our main findings, we incorporated Lasso logistic regression, a regularised version of logistic regression, which is useful for feature selection. Additionally, we conducted logistic regression models based on the EQD scheme to evaluate our primary analysis across comparable dose levels.

## 3 Results

**Figure 1:**
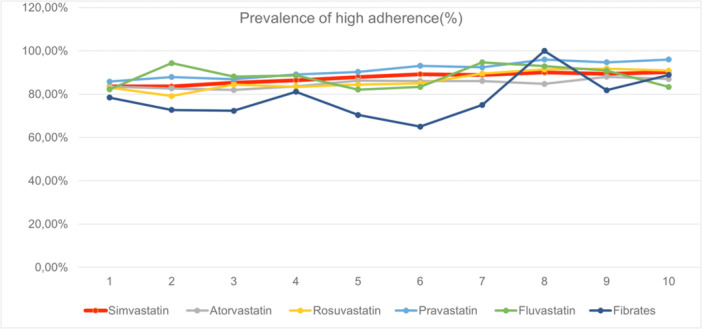
Prevalence of high adherence (the proportion of high adherence ≥ 0.8) from the 1st to the 10th year for patients who maintained antihyperlipidemic monotherapy for more than 1–10 years (adherence = covered days in each year/360 days; prevalence of high adherence = patients with adherence ≥ 0.8/all the patients).

Of the 18,375 initiators at baseline, 13,270 patients had a follow‐up time greater than 360 days (approx. 1 year), with an average adherence of 0.90 (SD 0.14) and prevalence of high adherence of 83.6% (see Supporting Information S1: Table S1). The baseline characteristics for patients on original antihyperlipidemic monotherapy exceeding 1 year see Supporting Information S1: Table S2. The prevalence of high adherence showed a slightly increasing trend from the 1st year to the 10th year. Similar trends can be seen among different anti‐hyperlipidemic monotherapies. Pravastatin showed the highest prevalence from the beginning to the end of follow up (85.8%–96.0%), followed by simvastatin (83.6%–90.2%), the prevalence of fibrates had the most complex and fluctuating changes (see Figure 1).

**Figure 2:**
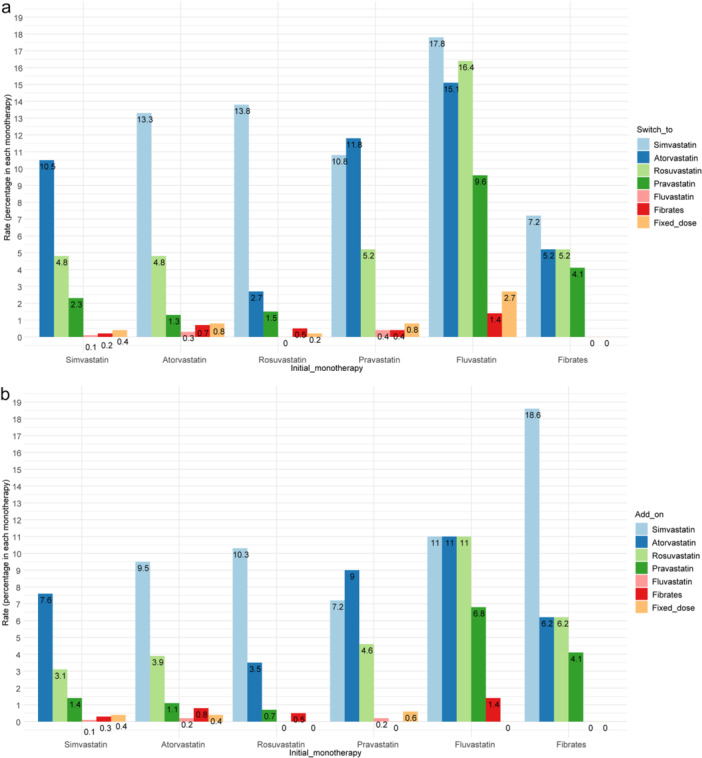
Switch and add on patterns of 6 classes of antihyperlipidemic monotherapy users in patients on original antihyperlipidemic monotherapy exceeding 1 year. (a) Rate of six antihyperlipidemic drug monotherapy switched to other drugs. (b) Rate of six antihyperlipidemic drug monotherapy added on other drugs. *Note:* The rate was the people switching to or add on different drugs among each monotherapy user.

In patients with follow‐up time greater than 1 year, simvastatin users were more likely to maintain the original monotherapy, were less likely to discontinue, and less likely to switch or add on other monotherapies or fixed‐dose combinations. Fluvastatin users were more likely to discontinue and change the monotherapy, and showed less continuation (Table 2). The most common drug categories were switched to simvastatin and atorvastatin, and were more likely to add simvastatin and atorvastatin as another drug (see Figure 2).

**Table 2:** General drug patterns in patients on original antihyperlipidemic monotherapy exceeding 1 year of use. *N* (%).

Drug pattern	Overall (*N* = 13270)	Simvastatin (*N* = 10475)	Atorvastatin (*N* = 1530)	Rosuvastatin (*N* = 594)	Pravastatin (*N* = 501)	Fluvastatin (*N* = 73)	Fibrates (*N* = 97)
Continuation	5044 (38.0%)	4109 (39.2%)	540 (35.3%)	218 (36.7%)	145 (28.9%)	8 (11.0%)	24 (24.7%)
Discontinuation	8090 (61.0%)	6294 (60.1%)	958 (62.6%)	363 (61.1%)	348 (69.5%)	64 (87.7%)	63 (64.9%)
Switch	2461 (18.5%)	1838 (17.5%)	309 (20.2%)	109 (18.4%)	141 (28.1%)	44 (60.3%)	20 (20.6%)
Add on	1784 (13.4%)	1299 (12.4%)	233 (15.2%)	87 (14.6%)	104 (20.8%)	30 (41.1%)	31 (32.0%)

Based on the EQD scheme (see Supporting Information S1: Table S3), most patients at baseline were at low risk since few patients used a drug monotherapy with high dose level and no patients were recorded using 80 mg simvastatin or atorvastatin per day. Most patients were more easily switched to or added medium EQD levels of atorvastatin or high EQD levels of rosuvastatin (Supporting Information S1: Tables S4–S5).

Table 3 showed the results of the multivariable logistic regression analysis of high adherence and drug patterns. The following statements were results compared to the reference group: Patients aged 40–69 years and greater than 70 years and with drug treatment for diabetes were positively associated with high adherence [OR and 95% CI were 1.68 (1.44–1.95), 2.46 (1.99–3.04), and 1.17 (1.05–1.29), respectively], whereas being male was negatively associated with high adherence (0.79, 0.72–0.87). When continuation was the outcome, pravastatin, fluvastatin and fibrate users and patients on antiparkinson drugs were less likely to continue treatment [0.79 (0.65–0.97), 0.25 (0.11–0.5), 0.58 (0.36–0.92), 0.59 (0.37–0.92)]; high adherence, being male, 40–69, older than 70 age groups, patients on diabetes drug and calendar year after 2010 were positively associated with good continuation [1.95 (1.76–2.16), 1.11 (1.03–1.2), 1.59 (1.38–1.84), 1.36 (1.14–1.62), 1.15 (1.06–1.24), 1.57 (1.31–1.89)]. When discontinuation was the outcome, fluvastatin users, patients on antiparkinson drug, calendar year between 2000 and 2010 were associated with higher discontinuation rate [3.66 (1.9–7.94), 1.66 (1.07–2.64), 1.22 (1.02–1.46)]. High adherence, being male, 40–69, older than 70 age groups, patients on diabetes drug and calendar year between 2010 and 2020 were negatively associated with discontinuation [0.5 (0.45–0.56), 0.9 (0.84–0.97), 0.62 (0.54–0.72), 0.75 (0.63–0.89), 0.87 (0.81–0.94), 0.66 (0.55–0.79)]. When switching was the outcome, pravastatin and fluvastatin users, high adherence, 40–69 age group was associated with increased switching rate [1.58 (1.29–1.94), 5.57 (3.46–9.08), 1.26 (1.12–1.44), 1.31 (1.1–1.57)] while patients on diabetes drug and calendar year between 2010 and 2020 were negatively associated with switching [0.75 (0.68–0.83), 0.69 (0.56–0.85)]. When add‐on was the outcome, pravastatin, fluvastatin and fibrate users, high adherence were associated with higher add‐on rates [1.61 (1.28–2.02), 4.06 (2.49–6.53), 3.13 (2–4.81), 1.47 (1.27–1.71)], while patients on diabetes drug and calendar year between 2010 and 2020 lowered the risk [0.81 (0.73–0.91), 0.74 (0.59–0.93)]. Most of the prediction models had a good fit according to the Hosmer‐Lemeshow test.

**Table 3:** Multivariable logistic regression analysis of risk factors for high adherence and drug patterns in patients on original antihyperlipidemic monotherapy exceeding 1 year.

	High adherence		Continuation		Discontinuation		Switch		Add on	
	OR (95% CI)	*p*	OR (95% CI)	*p*	OR (95% CI)	*p*	OR (95% CI)	*p*	OR (95% CI)	*p*
Antihyperlipidemic drug class										
Atorvastatin	1.04 (0.9–1.21)	0.575	1.06 (0.94–1.19)	0.365	0.9 (0.8–1.01)	0.062	1.02 (0.89–1.17)	0.797	1.11 (0.95–1.3)	0.184
Fibrates	0.84 (0.53–1.42)	0.500	0.58 (0.36–0.92)	0.025	1.06 (0.7–1.65)	0.785	1.14 (0.67–1.84)	0.606	3.13 (2–4.81)	< 0.001
Fluvastatin	0.93 (0.52–1.78)	0.810	0.25 (0.11–0.5)	< 0.001	3.66 (1.9–7.94)	< 0.001	5.57 (3.46–9.08)	< 0.001	4.06 (2.49–6.53)	< 0.001
Pravastatin	1.17 (0.91–1.53)	0.228	0.79 (0.65–0.97)	0.025	1.21 (0.99–1.48)	0.059	1.58 (1.29–1.94)	< 0.001	1.61 (1.28–2.02)	< 0.001
Rosuvastatin	1 (0.8–1.25)	0.980	1.07 (0.89–1.27)	0.462	0.88 (0.74–1.05)	0.145	0.94 (0.76–1.17)	0.592	1.1 (0.86–1.38)	0.447
Adherence: High	**/**	**/**	1.95 (1.76–2.16)	< 0.001	0.5 (0.45–0.56)	< 0.001	1.26 (1.12–1.44)	< 0.001	1.47 (1.27–1.71)	< 0.001
Sex: Male	0.79 (0.72–0.87)	< 0.001	1.11 (1.03–1.2)	0.004	0.9 (0.84–0.97)	0.004	0.95 (0.87–1.04)	0.305	0.97 (0.88–1.08)	0.600
Age (year)										
40–69	1.68 (1.44–1.95)	< 0.001	1.59 (1.38–1.84)	< 0.001	0.62 (0.54–0.72)	< 0.001	1.31 (1.1–1.57)	0.002	1.11 (0.92–1.35)	0.279
≥ 70	2.46 (1.99–3.04)	< 0.001	1.36 (1.14–1.62)	< 0.001	0.75 (0.63–0.89)	0.001	0.88 (0.7–1.1)	0.252	0.78 (0.61–1)	0.052
Baseline drug‐treated comorbidities										
Diabetes drug: Yes	1.17 (1.05–1.29)	0.003	1.15 (1.06–1.24)	< 0.001	0.87 (0.81–0.94)	< 0.001	0.75 (0.68–0.83)	< 0.001	0.81 (0.73–0.91)	< 0.001
RA drug: Yes	0.95 (0.59–1.6)	0.838	1.21 (0.82–1.76)	0.333	0.84 (0.57–1.22)	0.355	0.8 (0.46–1.32)	0.414	0.62 (0.3–1.13)	0.149
Asthma/COPD drug: Yes	1.16 (0.97–1.41)	0.114	1.08 (0.94–1.24)	0.266	0.93 (0.82–1.07)	0.326	1.01 (0.85–1.2)	0.914	1.15 (0.95–1.38)	0.141
Antiepileptics drug: Yes	1.25 (0.92–1.73)	0.173	0.92 (0.73–1.16)	0.479	1.09 (0.86–1.37)	0.483	1.03 (0.77–1.36)	0.823	1.32 (0.98–1.77)	0.064
Antiparkinson drug: Yes	1.04 (0.61–1.88)	0.898	0.59 (0.37–0.92)	0.024	1.66 (1.07–2.64)	0.026	0.59 (0.29–1.06)	0.101	0.73 (0.35–1.35)	0.357
Psycholeptics drug: Yes	0.9 (0.78–1.03)	0.123	0.91 (0.82–1.01)	0.091	1.09 (0.98–1.21)	0.123	0.94 (0.82–1.07)	0.363	0.95 (0.82–1.1)	0.510
Psychoanaleptic drug: Yes	1.15 (0.99–1.34)	0.069	0.9 (0.8–1.01)	0.076	1.11 (0.99–1.25)	0.067	1.03 (0.89–1.18)	0.706	1.12 (0.96–1.31)	0.142
Addictive_disorders drug: Yes	0.71 (0.44–1.2)	0.175	0.85 (0.54–1.3)	0.455	1.22 (0.8–1.91)	0.359	1.16 (0.68–1.91)	0.562	1.62 (0.93–2.67)	0.074
Antineoplastic drug: Yes	0.46 (0.22–1.05)	0.049	0.47 (0.19–1.01)	0.068	1.87 (0.88–4.33)	0.118	0.73 (0.22–1.89)	0.562	1.44 (0.48–3.49)	0.461
Calendar year										
2000–2010	0.91 (0.72–1.14)	0.425	0.83 (0.7–1)	0.052	1.22 (1.02–1.46)	0.030	1.01 (0.83–1.24)	0.892	1.05 (0.83–1.32)	0.705
2010–2020	0.91 (0.72–1.14)	0.428	1.57 (1.31–1.89)	< 0.001	0.66 (0.55–0.79)	< 0.001	0.69 (0.56–0.85)	< 0.001	0.74 (0.59–0.93)	0.010
AUC	0.5		0.558		0.558		0.505		0.5	
Sensitivity	—		0.83		0.3		1		—	
Specificity	—		0.28		0.82		0.01		—	
Hosmer‐Lemeshow test	*p* < 0.001		*p* = 0.111		*p* = 0.060		*p* = 0.175		*p* = 0.014	

*Note:* The reference groups of antihyperlipidemic drug class, adherence, sex, age, baseline drug‐treated comorbidities and calendar year were simvastatin, low adherence, female, 18–39 age group, without baseline drug‐treated comorbidities and 1996–2000 calendar year, respectively. /: Not included in the model. —: No relevant data available.

After IPW adjustments for other risk factors, fluvastatin and fibrates showed a relatively complex curve of the probability of staying in low adherence in 3‐year follow‐up time (see Supporting Information S1: Figure S1). All other drug monotherapies had a similar likelihood of achieving high adherence compared to simvastatin, both with and without time‐dependent effects (see Table 4).

**Table 4:** Cox regression analysis of high‐adherence (7303 patients on original antihyperlipidemic monotherapy ≤ 3 years in 15,470 patients with all drug records exceed 3 years).

	Without time‐dependent effects			
Antihyperlipidemic monotherapies	Crude HR (95% CI)	*p* value	IPW adjusted^a^ HR (95% CI)	*p* value
Reference: Simvastatin				
Drug treatment:				
Atorvastatin	0.96 (0.88–1.04)	0.302	1.03 (0.93–1.15)	0.573
Rosuvastatin	0.83 (0.73–0.96)	0.009	0.90 (0.77–1.06)	0.202
Pravastatin	0.88 (0.77–0.99)	0.040	0.92 (0.78–1.08)	0.299
Fluvastatin	0.95 (0.71–1.28)	0.753	0.60 (0.29–1.26)	0.177
Fibrates	0.77 (0.56–1.05)	0.097	0.83 (0.55–1.24)	0.355

	**With time‐dependent effects**			
Treatment	**Crude HR (95% CI)**	*p* value	**IPW adjusted** a **HR (95% CI)**	*p* value
Atorvastatin	1.34 (1.07–1.67)	0.010	1.29 (0.93–1.79)	0.134
Rosuvastatin	22.99 (3.06–172.72)	0.002	8.01 (0.38–169.11)	0.181
Pravastatin	2.33 (1.27–4.29)	0.006	1.75 (0.69–4.41)	0.235
Fluvastatin	1.85 (1.12–3.04)	0.015	0.92 (0.46–1.85)	0.816
Fibrates	1.39 (0.86–2.25)	0.175	1.22 (0.58–2.53)	0.601
tt (antihyperlipidemic drug monotherapy)	0.93 (0.89–0.97)	0.001	0.95 (0.89–1.02)	0.163

a IPW adjusted between antihyperlipidemic monotherapies and sex, age, drugs for diabetes, RA, asthma/COPD, antiepileptics, antiparkinson, psycholeptics, psychoanaleptic, addictive_disorders, antineoplastic, calendar‐year periods.

After the variables were filtered by additional Lasso logistic regression analyses, the potential risk factors showed similar patterns of high adherence and studied drug patterns, but the evaluation indicators (AUC) of the models improved (see Supporting Information S1: Table S6). The logistic regression analysis among different dosage of monotherapy at low level and medium level of EQD also showed that, 10 mg simvastatin and 20 mg simvastatin had better continuation probability and a lower probability to switch or add other monotherapy compared to equivalent doses of other monotherapy, especially in the low level of EQD comparison group (see Supporting Information S1: Table S7).

## 4 Discussion

The mean/average adherence in patients whose follow‐up time was greater than 1 year exceeded 0.8, a figure comparable to that found in a study of the US veteran population, which had an average age of 62 years and consisted mostly of middle‐aged men [18]. In that study, adherence rates were higher for pravastatin than for simvastatin, which is similar to our findings, though the difference was small. In contrast, another study that used pharmacy and medical claims from a US health plan database showed that patients starting in the simvastatin cohort were more adherent than those in the atorvastatin cohort (43% vs. 36%, respectively) [19]. In our study, there appeared not so much difference between these two monotherapies. This may be explained by the fact that study patients in our cohort had at least 1‐year follow up time which obviously raised the threshold for adherence and second, most of them used medium level doses of simvastatin or atorvastatin at baseline. Furthermore, the low average adherence or low prevalence of high adherence of fibrates may have three reasons: (1) guidelines suggest to use this drug as second‐line treatment [20], the health care providers only consider second‐line if first‐line failure, intolerance, or contraindications, so the cost or the negative perception from patients may contribute to lower adherence (2) a lower efficacy compared to statins [21] (3) complicated dosing schedule, such as the requirement to take it before meals, contrasts with statin therapy, which can be taken at any time of the day [22].

Many studies [4] suggest that patients use high‐dose statins. In our study, when patients chose to change their initial drug patterns—either by switching to or adding a new monotherapy or fixed‐dose combination—they were still more frequently prescribed a medium dose of atorvastatin rather than a high dose of rosuvastatin. Therefore, increasing the dosage may not always be the optimal choice. Additionally, prescribers tended to favour commonly used statins such as simvastatin and atorvastatin.

Male patients demonstrated lower adherence compared to female, which contrasted with traditional risk factors for adherence [23], but aligns with the findings of several studies summarised in a meta‐analysis [7]. Patients older than 70 years may have more time to focus on their health conditions, which could explain their better adherence and continued use of the initial drug monotherapy, as observed in the US study [18]. One study showed that patients with a history of diabetes exhibited higher adherence [24]. This can be understood as patients with multiple conditions being more attentive to their health. Additionally, they may receive more monitoring or notifications to remind them to take their medication. Furthermore, statins can prevent CVD especially in diabetes patients [25], although studies have reported contradictory results [18]. High adherence prevented discontinuation and facilitated continuation. Once patients have a lower drug adherence, the effectiveness is reduced which will lead to an unhealthy condition and this may lead to discontinuation. So increasing adherence is vital to the continuation of monotherapy assignment. Antiparkinson drug were associated with lower continuation rates of anti‐hyperlipidemic drug, it might due to the drug interactions [26], antiparkinson medications, such as levodopa or dopamine agonists, when used together with statins like rosuvastatin may increase the risk of nerve damage.

Most monotherapy users switched or added another drug more easily compared to simvastatin users. This may be explained by a higher incidence of perceived side effects or intolerance leading to treatment modification among users of other statins. The safety profile of statins is complex and context‐dependent. While a meta‐analysis showed that within statin classes, simvastatin and pravastatin demonstrated a higher level of safety and tolerability when compared to other statins [27]. One US study showed that Simvastatin‐associated reports showed signals for higher objective muscular adverse effects relative to all other statins [28]. This apparent discrepancy may reflect differences in study design, populations, or the significant role of the nocebo effect, whereby a substantial proportion of statin‐associated symptoms are driven by patient expectations rather than direct pharmacological toxicity [29]. The Netherlands cardiovascular management guideline also suggest that pravastatin is only recommended if the use of atorvastatin, rosuvastatin and simvastatin is not suitable due to side effects or the risk of rhabdomyolysis in interaction with other agents. Fluvastatin alone is recommended given its relatively high cost and weaker lipid lowering if other statins are not tolerated [12].

The relative likelihood of maintaining in low adherence or achieving high adherence did not differ significantly between the various monotherapies compared with simvastatin, and time‐dependent effects did not appear to influence the hazards over follow‐up. This finding suggests that, regardless of the specific anti‐hyperlipidemic monotherapy, patients showed similar patterns of adherence over time, with no substantial differences in either the short‐ or long‐term use. Therefore, when selecting personalised medication, healthcare providers should be aware that the choice of monotherapy may not substantially influence patients’ likelihood of achieving high adherence.

## 5 Strength and Limitations

The strengths of this study are its large cohort size and large number of participants, resulting in more reliable and generalisable findings for the Netherlands patient population. In addition, this study involved new users of treatment, providing the opportunity to track outcomes since initiation. The extended follow‐up period ensures that long‐term effects and outcomes can be observed, providing a more complete picture of the impact of the treatment. In addition, the study provides detailed and comprehensive information about the drug treatment, including precise analyses of dosage and adherence, thereby increasing the validity of the findings.

There were also some potential limitations to our study. The number of patients who used simvastatin was relatively large as compared to other monotherapies. However, this is according to the guidelines. Although IADB.nl database has complete records, medications dispensed during hospitalisations are absent. However, most studied therapies are dispensed from community pharmacies.

We lacked information regarding the lifestyle or socioeconomic status of patients, which are also factors important to predict adherence and prescription patterns [30, 31]. For example, many social and economic factors are associated with non‐adherence and such factors would indirectly promote the discontinuation. Further, the AUC values of prediction models were rather low, though the sensitivity analysis improved the AUC and the results remained similar to the main results. Finally, based on previous studies and the current baseline equivalent doses of each anti‐hyperlipidemic monotherapy, though the patients might have similar baseline cholesterol levels, the clinical characteristics were different, which means that the difference in the adherence, discontinuation, continuation, switch and add on were not only attributed to the exposure themselves but also pre‐existing differences in patient populations, which could influence the generalisability of the results. Furthermore, our findings in low‐risk primary prevention may not directly generalise to settings where potency‐driven treatment escalation is more common.

## 6 Conclusion

Simvastatin users demonstrated higher adherence and continuation rates compared to other anti‐hyperlipidemic monotherapy users. Key factors associated with high adherence included older age, female sex, and use of diabetes medications. For high continuation rates and lower discontinuation rates, predictors included older age, male sex, high adherence, use of diabetes medications, absence of antiparkinson drugs, and more recent calendar year. Conversely, low adherence, use of diabetes medications, recent calendar year, and/or younger age were linked to lower switch and add‐on rates. Sensitivity analyses confirmed the robustness of these findings. Understanding these risk factors is critical for developing personalised strategies to optimise CVD prevention.

## Author Contributions

X.C.L. conceived the study. S.M., E.H., C.C.M.S.V., and J.H.J. designed the study. J.H.J. constructed data. S.M. provided statistical support. X.C.L., S.M., and E.H. wrote the first draft. All the authors reviewed and approved the final article.

## Ethics Statement

This study is based on the University of Groningen IADB.nl database established and researched since 1995. Data were collected in accordance with current national and European guidelines on privacy requirements for handling human data. The authors have no ethical conflicts to disclose. Ethical approval was not required for this study.

## Consent

The authors have nothing to report.

## Conflicts of Interest

The authors declare no conflicts of interest.

## Supporting information

Supplementary_Information.

## Data Availability

The study remains in progress and the data are not currently available for sharing. All R code is available from the authors upon request.
