# Supplementary material for: Long‐Term Adherence and Drug Utilisation Patterns Among New Users of Anti‐Hyperlipidemic Monotherapy: Development of a Risk Prediction Model
Source: J Eval Clin Pract. 2026 Feb 18;32(1):e70380. doi: 10.1111/jep.70380 (PMC12917296; doi:10.1111/jep.70380)
Supplement: Supplementary file 1 — Supplementary_Information. [file JEP-32-0-s001.docx]

**Long-term Adherence and Drug Utilization Patterns Among New Users of Anti-hyperlipidemic Monotherapy: Development of a Risk Prediction Model**

**Running head:** Adherence and Drug Patterns of Anti-hyperlipidemic monotherapy

Xuechun Li ^1^, Eelko Hak ^1^, Jens H J Bos ^1^, Catharina C. M. Schuiling-Veninga ^1^, Sumaira Mubarik ^1^

^1^University of Groningen, unit PharmacoTherapy, -Epidemiology and -Economics, Groningen Research Institute of Pharmacy, 9713 AV Groningen, The Netherlands

Correspondence: Xuechun Li, PhD research fellow

PharmacoTherapy, -Epidemiology and -Economics, Groningen Research Institute of Pharmacy, University of Groningen, 9713 AV Groningen, The Netherlands

Tel : +31 64 901 9602 (mobile)

Email : xuechen.li@rug.nl

Eelko Hak: e.hak@rug.nl

Jens H J Bos: h.j.bos@rug.nl

Catharina C. M. Schuiling-Veninga: c.c.m.schuiling-veninga@rug.nl

Sumaira Mubarik: s.mubarik@rug.nl

**ORCID**

Xuechun Li, 0000-0003-2175-8307

Eelko Hak, 0000-0003-0849-7210

Jens H J Bos, 0000-0001-9209-7564

Catharina C. M. Schuiling-Veninga, 0000-0002-8527-2608

Sumaira Mubarik, 0000-0001-6041-1061

**Supplementary table 1.** Adherence description in patients on original antihyperlipidemic monotherapy exceeding 1 year to 10 years.

|  | **Average adherence** | | | | | | | **Prevalence of high adherence** |  |  |  |  |  |  |
| --- | --- | --- | --- | --- | --- | --- | --- | --- | --- | --- | --- | --- | --- | --- |
|  | overall | Simvastatin | Atorvastatin | Rosuvastatin | Pravastatin | Fluvastatin | Fibrates | overall | Simvastatin | Atorvastatin | Rosuvastatin | Pravastatin | Fluvastatin | Fibrates |
| 1-year, n=13270 | 0.90 (0.14) | 0.90 (0.14) | 0.90 (0.14) | 0.90 (0.15) | 0.91 (0.13) | 0.91 (0.12) | 0.89 (0.13) | 11093 (83.6%) | 8756 (83.6%) | 1278 (83.5%) | 493 (83.0%) | 430 (85.8%) | 60 (82.2%) | 76 (78.4%) |
| 2-year, n=10292 | 0.90 (0.14) | 0.90 (0.14) | 0.90 (0.15) | 0.87 (0.19) | 0.91 (0.12) | 0.93 (0.10) | 0.86 (0.17) | 8585 (83.4%) | 6820 (83.6%) | 967 (82.6%) | 359 (79.1%) | 341 (87.9%) | 50 (94.3%) | 48 (72.7%) |
| 3-year, n=8167 | 0.91 (0.14) | 0.91 (0.13) | 0.89 (0.16) | 0.90 (0.16) | 0.92 (0.13) | 0.93 (0.10) | 0.85 (0.21) | 6933 (84.9%) | 5513 (85.3%) | 791 (81.9%) | 293 (84.4%) | 265 (86.9%) | 37 (88.1%) | 34 (72.3%) |
| 4-year, n=6606 | 0.91 (0.13) | 0.92 (0.13) | 0.90 (0.17) | 0.90 (0.14) | 0.92 (0.12) | 0.92 (0.12) | 0.87 (0.18) | 5682 (86.0%) | 4510 (86.4%) | 664 (83.6%) | 226 (83.4%) | 221 (89.1%) | 31 (88.6%) | 30 (81.1%) |
| 5-year, n=5380 | 0.92 (0.12) | 0.92 (0.12) | 0.91 (0.16) | 0.90 (0.14) | 0.93 (0.10) | 0.89 (0.15) | 0.84 (0.26) | 4710 (87.5%) | 3740 (87.9%) | 562 (86.3%) | 179 (84.4%) | 187 (90.3%) | 23 (82.1%) | 19 (70.4%) |
| 6-year, n=4355 | 0.92 (0.12) | 0.93 (0.11) | 0.91 (0.16) | 0.91 (0.11) | 0.93 (0.11) | 0.90 (0.11) | 0.83 (0.21) | 3862 (88.7%) | 3083 (89.2%) | 453 (86.1%) | 130 (85.0%) | 163 (93.1%) | 20 (83.3%) | 13 (65.0%) |
| 7-year, n=3547 | 0.92 (0.12) | 0.93 (0.11) | 0.90 (0.17) | 0.93 (0.11) | 0.93 (0.13) | 0.93 (0.07) | 0.90 (0.12) | 3146 (88.7%) | 2495 (88.9%) | 377 (86.1%) | 111 (89.5%) | 133 (92.4%) | 18 (94.7%) | 12 (75.0%) |
| 8-year, n=2833 | 0.93 (0.11) | 0.93 (0.11) | 0.90 (0.16) | 0.94 (0.10) | 0.94 (0.06) | 0.91 (0.12) | 0.94 (0.06) | 2540 (89.7%) | 1983 (90.0%) | 316 (84.7%) | 96 (91.4%) | 119 (96.0%) | 13 (92.9%) | 13 (100%) |
| 9-year, n=2322 | 0.93 (0.11) | 0.93 (0.10) | 0.91 (0.13) | 0.93 (0.10) | 0.94 (0.09) | 0.89 (0.16) | 0.93 (0.09) | 2078 (89.5%) | 1588 (89.4%) | 286 (88.0%) | 78 (91.8%) | 107 (94.7%) | 10 (90.9%) | 9 (81.8%) |
| 10-year, n=1864 | 0.94 (0.10) | 0.93 (0.10) | 0.93 (0.10) | 0.94 (0.08) | 0.95 (0.07) | 0.91 (0.10) | 0.93 (0.08) | 1679 (90.1%) | 1258 (90.2%) | 241 (87.0%) | 70 (90.9%) | 97 (96.0%) | 5 (83.3%) | 8 (88.9%) |

**Supplementary table 2.** Baseline characteristics for patients on original antihyperlipidemic monotherapy exceeding 1 year

| **Demographics** | **Overall** | **Simvastatin** | **Atorvastatin** | **Rosuvastatin** | **Pravastatin** | **Fluvastatin** | **Fibrates** |
| --- | --- | --- | --- | --- | --- | --- | --- |
|  | N=13270 | N=10475(78.9%)* | N=1530(11.5%)* | N=594(4.5%)* | N=501(3.8%)* | N=73(0.6%)* | N=97(0.7%)* |
| **Sex :Male** | 7456 (56.2%) | 5931 (56.6%) | 839 (54.8%) | 303 (51.0%) | 267 (53.3%) | 42 (57.5%) | 74 (76.3%) |
| **Age at entry† (years)** | 56.1 (11.4) | 56.5 (11.3) | 54.0 (11.7) | 55.1 (11.9) | 56.3 (11.5) | 54.6 (9.46) | 45.5 (9.72) |
| 18-39 | 1032 (7.8%) | 744 (7.1%) | 162 (10.6%) | 63 (10.6%) | 35 (7.0%) | 4 (5.5%) | 24 (24.7%) |
| 40-69 | 10662 (80.3%) | 8429 (80.5%) | 1231 (80.5%) | 466 (78.5%) | 400 (79.8%) | 65 (89.0%) | 71 (73.2%) |
| ≥70 | 1576 (11.9%) | 1302 (12.4%) | 137 (9.0%) | 65 (10.9%) | 66 (13.2%) | 4 (5.5%) | 2 (2.1%) |
| **Initial comorbidities drug use£** |  |  |  |  |  |  |  |
| Diabetes drug: yes | 4520 (34.1%) | 3791 (36.2%) | 371 (24.2%) | 140 (23.6%) | 177 (35.3%) | 16 (21.9%) | 25 (25.8%) |
| RA drug: yes | 117 (0.9%) | 98 (0.9%) | 12 (0.8%) | 4 (0.7%) | 2 (0.4%) | 0 (0%) | 1 (1.0%) |
| Asthma/COPD drug: yes | 967 (7.3%) | 788 (7.5%) | 100 (6.5%) | 43 (7.2%) | 27 (5.4%) | 2 (2.7%) | 7 (7.2%) |
| Antiepileptics drug: yes | 333 (2.5%) | 254 (2.4%) | 27 (1.8%) | 21 (3.5%) | 23 (4.6%) | 2 (2.7%) | 6 (6.2%) |
| Antiparkinson drug: yes | 100 (0.8%) | 84 (0.8%) | 12 (0.8%) | 1 (0.2%) | 3 (0.6%) | 0 (0%) | 0 (0%) |
| Psycholeptics drug: yes | 1983 (14.9%) | 1521 (14.5%) | 258 (16.9%) | 93 (15.7%) | 83 (16.6%) | 15 (20.5%) | 13 (13.4%) |
| Psychoanaleptics drug: yes | 1671 (12.6%) | 1336 (12.8%) | 191 (12.5%) | 68 (11.4%) | 59 (11.8%) | 6 (8.2%) | 11 (11.3%) |
| Addictive_disorders drug: yes | 93 (0.7%) | 77 (0.7%) | 8 (0.5%) | 3 (0.5%) | 4 (0.8%) | 1 (1.4%) | 0 (0%) |
| Antineoplastic drug: yes | 33 (0.2%) | 26 (0.2%) | 3 (0.2%) | 3 (0.5%) | 1 (0.2%) | 0 (0%) | 0 (0%) |
| **Calendar years** |  |  |  |  |  |  |  |
| 1996-2000 | 617 (4.6%) | 394 (3.8%) | 120 (7.8%) | 0 (0%) | 54 (10.8%) | 30 (41.1%) | 19 (19.6%) |
| 2000-2010 | 5731 (43.2%) | 3935 (37.6%) | 1000 (65.4%) | 366 (61.6%) | 353 (70.5%) | 40 (54.8%) | 37 (38.1%) |
| 2010-2020 | 6922 (52.2%) | 6146 (58.7%) | 410 (26.8%) | 228 (38.4%) | 94 (18.8%) | 3 (4.1%) | 41 (42.3%) |

* Row percentage, others are all column percentage.

**†** Use mean±SD to describe continuous age.

£ Baseline prescriptions of any of these diseases (180 days after index date)

**Supplementary table 3.** Three levels of different equivalent doses (EQD) scheme

| **Low level of dose** | 10mg_simvastatin (567) |
| --- | --- |
|  | 10mg_pravastatin (56) |
|  | 20mg_pravastatin (165) |
|  | 20mg_fluvastatin (37) |
|  | 40mg_fluvastatin (18) |
|  | Lowdose_Fibrates (95) |
| **Medium level of dose** | 20mg_simvastatin (3141) |
|  | 40mg_simvastatin (6347) |
|  | 60mg_simvastatin (2) |
|  | 10mg_atorvastatin (626) |
|  | 20mg_atorvastatin (609) |
|  | 5mg_rosuvastatin (121) |
|  | 40mg_pravastatin (277) |
|  | 80mg_pravastatin (0) |
|  | 80mg_fluvastatin (18) |
| **High level of dose** | 80mg_simvastatin (0) |
|  | 40mg_atorvastatin (196) |
|  | 80mg_atorvastatin (0) |
|  | 10mg_rosuvastatin (380) |
|  | 20mg_rosuvastatin (44) |
|  | 40mg_rosuvastatin (9) |

**Supplementary table 4.** Switch dose patters of EQD in patients on original antihyperlipidemic monotherapy exceeding 1 year

| **Baseline monotherapy (2263 patients switched to any of the 6 classes of monotherapy with EQD information available)** | **Switch rate** | **Switched monotherapy** | **Total switch rate** | **level** |
| --- | --- | --- | --- | --- |
| **Total patients switched to atorvastatin (N=1100)** | **48.6%** |  |  |  |
| 40mg_simvastatin (N=632) level=M | 57.5% | 10mg_atorvastatin  20mg_atorvastatin  40mg_atorvastatin  80mg_atorvastatin | 320 (29.1%)  416 (37.8%)  358 (32.5%)  6 (0.5%) | M  M  H  H |
| 20mg_simvastatin (N=313) level=M | 28.5% |  |  |  |
| 10mg_simvastatin (N=64) level=L | 5.8**%** |  |  |  |
| 40mg_pravastatin (N=39) level=M | 3.5% |  |  |  |
| 20mg_pravastatin (N=15) level=L | 1.4% |  |  |  |
| 10mg_rosuvastatin (N=10) level=H | 0.9**%** |  |  |  |
| 20mg_fluvastatin (N=6) level=L | 0.5% |  |  |  |
| 10mg_pravastatin (N=5) level=L | 0.5**%** |  |  |  |
| 40mg_fluvastatin (N=5) level=L | 0.5% |  |  |  |
| 5mg_rosuvastatin (N=5) level=M | 0.5% |  |  |  |
| lowdose_Fibrates (N=5) level=L | 0.5% |  |  |  |
| 20mg_rosuvastatin (N=1) level=H | 0.1% |  |  |  |
| **Total patients switched to rosuvastatin (N=572)** | **25.3%** |  |  |  |
| 40mg_simvastatin (N=270) level=M | 47.2% | 10mg_rosuvastatin  20mg_rosuvastatin  5mg_rosuvastatin  40mg_rosuvastatin | 231 (40.4%)  99 (17.3%)  222 (38.8%)  20 (3.5%) | H  H  M  H |
| 20mg_simvastatin (N=169) level=M | 29.5% |  |  |  |
| 20mg_atorvastatin (N=35) level=M | 6.1% |  |  |  |
| 10mg_atorvastatin (N=26) level=M | 4.5% |  |  |  |
| 10mg_simvastatin (N=17) level=L | 3.0% |  |  |  |
| 40mg_pravastatin (N=14) level=M | 2.4% |  |  |  |
| 40mg_atorvastatin (N=11) level=H | 1.9% |  |  |  |
| 20mg_pravastatin (N=9) level=L | 1.6% |  |  |  |
| 80mg_fluvastatin (N=6) level=M | 1.0% |  |  |  |
| lowdose_Fibrates (N=5) level=L | 0.9% |  |  |  |
| 20mg_fluvastatin (N=4) level=L | 0.7% |  |  |  |
| 10mg_pravastatin (N=3) level=L | 0.5% |  |  |  |
| 40mg_fluvastatin (N=2) level=L | 0.3% |  |  |  |
| 60mg_simvastatin (N=1) level=M | 0.2% |  |  |  |
| **Total patients switched to simvastatin** **(N=355)** | **15.7%** |  |  |  |
| 10mg_atorvastatin (N=108) level=M | 30.4**%** | 10mg_simvastatin  20mg_simvastatin  40mg_simvastatin  60mg_simvastatin  80mg_simvastatin | 29 (8.2%)  120 (33.8%)  202 (56.9%)  3 (0.8%)  1 (0.3%) | L  M  M  M  H |
| 20mg_atorvastatin (N=74) level=M | 20.8**%** |  |  |  |
| 10mg_rosuvastatin (N=62) level=H | 17.5**%** |  |  |  |
| 40mg_pravastatin (N=32) level=M | 9.0**%** |  |  |  |
| 40mg_atorvastatin (N=19) level=H | 5.4**%** |  |  |  |
| 20mg_pravastatin (N=16) level=L | 4.5**%** |  |  |  |
| 20mg_rosuvastatin (N=11) level=H | 3.1**%** |  |  |  |
| 20mg_fluvastatin (N=8) level=L | 2.3**%** |  |  |  |
| lowdose_Fibrates (N=7) level=L | 2.0**%** |  |  |  |
| 10mg_pravastatin (N=6) level=L | 1.7**%** |  |  |  |
| 5mg_rosuvastatin (N=6) level=M | 1.7**%** |  |  |  |
| 80mg_fluvastatin (N=3) level=M | 0.8**%** |  |  |  |
| 40mg_fluvastatin (N=2) level=L | 0.6**%** |  |  |  |
| 40mg_rosuvastatin (N=1) level=H | 0.3**%** |  |  |  |
| **Total patients switched to pravastatin (N=276)** | **12.2%** |  |  |  |
| 40mg_simvastatin (N=129) level=M | 46.7% | 10mg_pravastatin  20mg_pravastatin  40mg_pravastatin | 45 (16.3%)  107 (38.8%)  124 (44.9%) | L  L  M |
| 20mg_simvastatin (N=94) level=M | 34.1% |  |  |  |
| 10mg_simvastatin (N=13) level=L | 4.7% |  |  |  |
| 20mg_atorvastatin (N=11) level=M | 4.0% |  |  |  |
| 10mg_atorvastatin (N=8) level=M | 2.9% |  |  |  |
| 10mg_rosuvastatin (N=6) level=H | 2.2% |  |  |  |
| 20mg_fluvastatin (N=6) level=L | 2.2% |  |  |  |
| lowdose_Fibrates (N=4) level=L | 1.4% |  |  |  |
| 20mg_rosuvastatin (N=2) level=H | 0.7% |  |  |  |
| 40mg_atorvastatin (N=1) level=H | 0.4% |  |  |  |
| 40mg_fluvastatin (N=1) level=L | 0.4% |  |  |  |
| 5mg_rosuvastatin (N=1) level=M | 0.4% |  |  |  |
| **Total patients switched to fibrates (N=39)** | **1.7%** |  |  |  |
| 20mg_simvastatin (N=11) level=M | 28.2% | lowdose_Fibrates | 39 (100%) | L |
| 40mg_simvastatin (N=8) level=M | 20.5% |  |  |  |
| 10mg_simvastatin (N=4) level=L | 10.3% |  |  |  |
| 20mg_atorvastatin (N=4) level=M | 10.3% |  |  |  |
| 10mg_atorvastatin (N=3) level=M | 7.7% |  |  |  |
| 10mg_rosuvastatin (N=3) level=H | 7.7% |  |  |  |
| 40mg_atorvastatin (N=3) level=H | 7.7% |  |  |  |
| 10mg_pravastatin (N=1) level=L | 2.6% |  |  |  |
| 20mg_fluvastatin (N=1) level=L | 2.6% |  |  |  |
| 20mg_pravastatin (N=1) level=L | 2.6% |  |  |  |
| **Total patients switched to fluvastatin (N=19)** | **0.8%** |  |  |  |
| 40mg_simvastatin (N=7) level=M | 36.8% | 40mg_fluvastatin  80mg_fluvastatin  20mg_fluvastatin | 7 (36.8%)  6 (31.6%)  6 (31.6%) | L  M  L |
| 20mg_simvastatin (N=6) level=M | 31.6% |  |  |  |
| 20mg_atorvastatin (N=3) level=M | 15.8% |  |  |  |
| 20mg_pravastatin (N=2) level=L | 10.5% |  |  |  |
| 40mg_atorvastatin (N=1) level=H | 5.3% |  |  |  |

**Supplementary table 5.** Add-on dose patterns of EQD in in patients on original antihyperlipidemic monotherapy exceeding 1 year

| **Baseline monotherapy (1588 patients added any of the 6 classes of monotherapy with EQD information available)** | **Add on rate** | **Added monotherapy** | **Total add on rate** | **Level** |
| --- | --- | --- | --- | --- |
| **Total patients added atorvastatin (N=781)** | **49.2%** |  |  |  |
| 40mg_simvastatin (N=458) level=M | 41.6% | 10mg_atorvastatin  20mg_atorvastatin  40mg_atorvastatin | 171 (21.9%)  310 (39.7%)  300 (38.4%) | M  M  H |
| 20mg_simvastatin (N=208) level=M | 18.9% |  |  |  |
| 10mg_simvastatin (N=37) level=L | 3.4% |  |  |  |
| 40mg_pravastatin (N=27) level=M | 2.5% |  |  |  |
| 10mg_rosuvastatin (N=14) level=H | 1.3% |  |  |  |
| 20mg_pravastatin (N=13) level=L | 1.2% |  |  |  |
| lowdose_Fibrates (N=6) level=L | 0.5% |  |  |  |
| 20mg_fluvastatin (N=5) level=L | 0.5% |  |  |  |
| 5mg_rosuvastatin (N=5) level=M | 0.5% |  |  |  |
| 10mg_pravastatin (N=4) level=L | 0.4% |  |  |  |
| 40mg_fluvastatin (N=3) level=L | 0.3% |  |  |  |
| 20mg_rosuvastatin (N=1) level=H | 0.1% |  |  |  |
| **Total patients added rosuvastatin (N=363)** | **22.9%** |  |  |  |
| 40mg_simvastatin (N=153) level=M | 42.1% | 10mg_rosuvastatin  20mg_rosuvastatin  5mg_rosuvastatin  40mg_rosuvastatin | 165 (45.5%)  69 (19.0%)  117 (32.2%)  12 (3.3%) | H  H  M  H |
| 20mg_simvastatin (N=110) level=M | 30.3% |  |  |  |
| 20mg_atorvastatin (N=27) level=M | 7.4% |  |  |  |
| 10mg_atorvastatin (N=20) level=M | 5.5% |  |  |  |
| 40mg_pravastatin (N=12) level=M | 3.3% |  |  |  |
| 40mg_atorvastatin (N=9) level=H | 2.5% |  |  |  |
| 10mg_simvastatin (N=8) level=L | 2.2% |  |  |  |
| 20mg_pravastatin (N=7) level=L | 1.9% |  |  |  |
| 80mg_fluvastatin (N=6) level=M | 1.7% |  |  |  |
| lowdose_Fibrates (N=6) level=L | 1.7% |  |  |  |
| 10mg_pravastatin (N=2) level=L | 0.6% |  |  |  |
| 20mg_fluvastatin (N=2) level=L | 0.6% |  |  |  |
| 60mg_simvastatin (N=1) level=M | 0.3% |  |  |  |
| **Total patients added simvastatin** **(N=268)** | **16.9%** |  |  |  |
| 10mg_atorvastatin (N=85) level=M | 31.7% | 10mg_simvastatin  20mg_simvastatin  40mg_simvastatin  60mg_simvastatin  80mg_simvastatin | 19 (7.1%)  81 (30.2%)  166 (61.9%)  1 (0.4%)  1 (0.4%) | L  M  M  M  H |
| 10mg_rosuvastatin (N=46) level=H | 17.2% |  |  |  |
| 20mg_atorvastatin (N=43) level=M | 16.0% |  |  |  |
| 40mg_pravastatin (N=21) level=M | 7.8% |  |  |  |
| 40mg_atorvastatin (N=18) level=H | 6.7% |  |  |  |
| lowdose_Fibrates (N=18) level=L | 6.7% |  |  |  |
| 20mg_pravastatin (N=10) level=L | 3.7% |  |  |  |
| 20mg_fluvastatin (N=7) level=L | 2.6% |  |  |  |
| 20mg_rosuvastatin (N=7) level=H | 2.6% |  |  |  |
| 5mg_rosuvastatin (N=7) level=M | 2.6% |  |  |  |
| 10mg_pravastatin (N=5) level=L | 1.9% |  |  |  |
| 80mg_fluvastatin (N=1) level=M | 0.4% |  |  |  |
| **Total patients added pravastatin (N=170)** | **10.7%** |  |  |  |
| 40mg_simvastatin (N=76) level=M | 44.7% | 10mg_pravastatin  20mg_pravastatin  40mg_pravastatin | 22 (12.9%)  57 (33.5%)  91 (53.5%) | L  L  M |
| 20mg_simvastatin (N=55) level=M | 32.4% |  |  |  |
| 10mg_simvastatin (N=9) level=L | 5.3% |  |  |  |
| 20mg_atorvastatin (N=8) level=M | 4.7% |  |  |  |
| 10mg_atorvastatin (N=7) level=M | 4.1% |  |  |  |
| lowdose_Fibrates (N=4) level=L | 2.4% |  |  |  |
| 10mg_rosuvastatin (N=3) level=H | 1.8% |  |  |  |
| 20mg_fluvastatin (N=3) level=L | 1.8% |  |  |  |
| 40mg_atorvastatin (N=2) level=H | 1.2% |  |  |  |
| 40mg_fluvastatin (N=2) level=L | 1.2% |  |  |  |
| 5mg_rosuvastatin (N=1) level=M | 0.6% |  |  |  |
| **Total patients added fibrates (N=49)** | **3.1%** |  |  |  |
| 40mg_simvastatin (N=15) level=M | 30.6% | lowdose_Fibrates | 49 (100%) | L |
| 20mg_simvastatin (N=13) level=M | 26.5% |  |  |  |
| 10mg_atorvastatin (N=6) level=M | 12.2% |  |  |  |
| 10mg_simvastatin (N=4) level=L | 8.2% |  |  |  |
| 40mg_atorvastatin (N=4) level=H | 8.2% |  |  |  |
| 10mg_rosuvastatin (N=3) level=H | 6.1% |  |  |  |
| 20mg_atorvastatin (N=3) level=M | 6.1% |  |  |  |
| 40mg_fluvastatin (N=1) level=L | 2.0% |  |  |  |
| **Total patients added fluvastatin (N=14)** | **0.9%** |  |  |  |
| 20mg_simvastatin (N=6) level=M | 42.9% | 80mg_fluvastatin  20mg_fluvastatin  40mg_fluvastatin | 5 (35.7%)  8 (57.1%)  1 (7.1%) | M  L  L |
| 40mg_simvastatin (N=4) level=M | 28.6% |  |  |  |
| 20mg_atorvastatin (N=2) level=M | 14.3% |  |  |  |
| 20mg_pravastatin (N=1) level=L | 7.1% |  |  |  |
| 40mg_atorvastatin (N=1) level=H | 7.1% |  |  |  |

**Supplementary table 6.** Lasso Logistic regression analysis of risk factors for high adherence and drug patterns in patients on original antihyperlipidemic monotherapy exceeding 1 year

|  | **High adherence** |  | **Continuation** |  | **Discontinuation** |  | **Switch** |  | **Add on** |  |
| --- | --- | --- | --- | --- | --- | --- | --- | --- | --- | --- |
| **Antihyperlipidemic drug class** | OR (95% CI) | p | OR (95% CI) | p | OR (95% CI) | p | OR (95% CI) | p | OR (95% CI) | p |
| Atorvastatin | 1.04 (0.9~1.21) | 0.575 | 1.06 (0.94~1.19) | 0.367 | 0.9 (0.8~1.01) | 0.062 | 1.02 (0.88~1.17) | 0.802 | 1.11 (0.95~1.3) | 0.188 |
| Fibrates | 0.84 (0.53~1.41) | 0.499 | 0.58 (0.36~0.92) | **0.026** | 1.06 (0.69~1.65) | 0.787 | 1.13 (0.67~1.83) | 0.631 | 3.12 (1.99~4.79) | **<0.001** |
| Fluvastatin | 0.93 (0.52~1.78) | 0.810 | 0.25 (0.11~0.5) | **<0.001** | 3.67 (1.91~7.96) | **<0.001** | 5.56 (3.46~9.07) | **<0.001** | 4.04 (2.48~6.51) | **<0.001** |
| Pravastatin | 1.17 (0.91~1.53) | 0.228 | 0.79 (0.65~0.97) | **0.024** | 1.21 (1~1.48) | 0.058 | 1.59 (1.29~1.94) | **<0.001** | 1.61 (1.28~2.02) | **<0.001** |
| Rosuvastatin | 1 (0.8~1.25) | 0.979 | 1.07 (0.89~1.27) | 0.462 | 0.88 (0.74~1.05) | 0.145 | 0.94 (0.76~1.17) | 0.601 | 1.1 (0.86~1.38) | 0.443 |
| **Adherence:High** | **/** | **/** | 1.95 (1.76~2.17) | **<0.001** | 0.5 (0.45~0.56) | **<0.001** | 1.27 (1.12~1.44) | **<0.001** | 1.47 (1.27~1.72) | **<0.001** |
| **Sex** :Male | 0.79 (0.72~0.87) | **<0.001** | 1.11 (1.03~1.2) | **0.005** | 0.9 (0.84~0.97) | **0.005** | - | **-** | - | - |
| **Age (year)** |  |  |  |  |  |  |  |  |  |  |
| 40-69 | 1.68 (1.44~1.95) | **<0.001** | 1.59 (1.38~1.84) | **<0.001** | 0.62 (0.54~0.72) | **<0.001** | 1.32 (1.11~1.58) | **0.002** | 1.11 (0.92~1.36) | 0.270 |
| ≥70 | 2.46 (1.99~3.04) | **<0.001** | 1.36 (1.14~1.62) | **<0.001** | 0.75 (0.63~0.89) | **0.001** | 0.88 (0.7~1.1) | 0.265 | 0.78 (0.61~1) | 0.054 |
| **Baseline drug-treated comorbidities** |  |  |  |  |  |  |  |  |  |  |
| **Diabetes drug:** Yes | 1.17 (1.06~1.29) | **0.003** | 1.15 (1.06~1.24) | **<0.001** | 0.87 (0.81~0.94) | **<0.001** | 0.75 (0.68~0.83) | **<0.001** | 0.81 (0.73~0.9) | **<0.001** |
| **RA drug:** Yes | - | - | 1.21 (0.83~1.76) | 0.324 | 0.83 (0.57~1.22) | 0.346 | - | - | 0.62 (0.3~1.14) | 0.154 |
| **Asthma/COPD drug:** Yes | 1.16 (0.97~1.41) | 0.115 |  |  |  |  | - | - | 1.15 (0.95~1.38) | 0.143 |
| **Antiepileptics drug:** Yes | 1.25 (0.92~1.73) | 0.173 | 0.92 (0.72~1.16) | 0.469 | 1.09 (0.86~1.38) | 0.475 | - | - | 1.31 (0.97~1.75) | 0.071 |
| **Antiparkinson drug:** Yes | - | - | 0.6 (0.37~0.93) | **0.025** | 1.66 (1.07~2.63) | **0.027** | 0.59 (0.3~1.07) | 0.107 | 0.72 (0.35~1.33) | 0.331 |
| **Psycholeptics drug:** Yes | 0.9 (0.78~1.03) | 0.125 | 0.91 (0.82~1.02) | 0.099 | 1.09 (0.98~1.21) | 0.132 | 0.96 (0.84~1.09) | 0.507 | - | - |
| **Psychoanaleptic drug:** Yes | 1.15 (0.99~1.34) | 0.069 | 0.9 (0.8~1.01) | 0.077 | 1.11 (0.99~1.25) | 0.067 | - | - | 1.11 (0.95~1.29) | 0.170 |
| **Addictive_disorders drug:** Yes | 0.71 (0.44~1.2) | 0.176 | 0.85 (0.55~1.3) | 0.462 | 1.22 (0.8~1.9) | 0.364 | - | - | 1.6 (0.92~2.65) | 0.079 |
| **Antineoplastic drug:** Yes | 0.45 (0.22~1.04) | **0.044** | 0.47 (0.2~1.02) | 0.070 | 1.86 (0.88~4.31) | 0.120 | - | - | 1.43 (0.48~3.48) | 0.467 |
| **Calendar year** |  |  |  |  |  |  |  |  |  |  |
| 2000-2010 | 0.91 (0.72~1.14) | 0.425 | 0.83 (0.7~1) | 0.053 | 1.22 (1.02~1.46) | **0.031** | 1.01 (0.83~1.24) | 0.912 | 1.05 (0.84~1.32) | 0.704 |
| 2010-2020 | 0.91 (0.72~1.14) | 0.426 | 1.58 (1.32~1.89) | **<0.001** | 0.66 (0.55~0.79) | **<0.001** | 0.69 (0.56~0.85) | **<0.001** | 0.74 (0.59~0.93) | **0.010** |
| **AUC** | 0.542 |  | 0.615 |  | 0.614 |  | 0.588 |  | 0.570 |  |
| **Sensitivity** | 1 |  | 0.134 |  | 0.821 |  | 0.013 |  | 0 |  |
| **Specificity** | 0 |  | 0.923 |  | 0.294 |  | 0.998 |  | 1 |  |
| **Hosmer-Lemeshow test** | P<0.001 |  | P=0.027 |  | P=0.037 |  | **P=0.078** |  | **P=0.1** |  |

The reference groups of antihyperlipidemic drug class, adherence, sex, age, baseline drug-treated comorbidities and calendar year were simvastatin, low adherence, female, 18-39 age group, without baseline drug-treated comorbidities and 1996-2000 calendar year respectively.

/: not included in the model

-: No relevant data available.

**Supplementary Table 7**. Multivariable Logistic regression analysis of EQD for high adherence and drug patterns in patients on original antihyperlipidemic monotherapy exceeding 1 year

|  | **High adherence** |  | **Continuation** |  | **Discontinuation** |  | **Switch** |  | **Add on** |  |
| --- | --- | --- | --- | --- | --- | --- | --- | --- | --- | --- |
|  | **OR (95% CI)** | **P** | **OR (95% CI)** | **P** | **OR (95% CI)** | **p** | **OR (95% CI)** | **p** | **OR (95% CI)** | **p** |
| **Antihyperlipidemic drug EQD** |  |  |  |  |  |  |  |  |  |  |
| **Low level comparison** |  |  |  |  |  |  |  |  |  |  |
| **10mg_simvastatin (567)** | 1 |  | 1 |  | 1 |  | 1 |  | 1 |  |
| 10mg_pravastatin (56) | 1.32 (0.59~3.53) | 0.541 | 0.46 (0.23~0.86) | **0.019** | 2.21 (1.19~4.4) | **0.017** | 1.77 (0.91~3.27) | 0.079 | 1.9 (0.89~3.77) | 0.076 |
| 20mg_pravastatin (165) | 1.11 (0.67~1.91) | 0.703 | 0.92 (0.64~1.33) | 0.664 | 1.04 (0.72~1.51) | 0.826 | 1.66 (1.08~2.52) | **0.018** | 1.7 (1.04~2.74) | **0.030** |
| 20mg_fluvastatin (37) | 0.52 (0.24~1.23) | 0.112 | 0.21 (0.06~0.53) | **0.003** | 3.88 (1.61~11.56) | **0.006** | 8.4 (4.17~17.67) | **<0.001** | 6.55 (3.21~13.27) | **<0.001** |
| 40mg_fluvastatin (18) | 0.83 (0.26~3.65) | 0.772 | 0.09 (0.01~0.46) | **0.021** | 10.98 (2.22~199.04) | **0.020** | 6.94 (2.65~19.37) | **<0.001** | 3.64 (1.22~9.8) | **0.013** |
| Lowdose_Fibrates (95) | 0.75 (0.43~1.35) | 0.320 | 0.51 (0.3~0.83) | **0.008** | 1.19 (0.75~1.9) | 0.467 | 1.41 (0.8~2.4) | 0.216 | 4.07 (2.43~6.75) | **<0.001** |
| **Medium level comparison** |  |  |  |  |  |  |  |  |  |  |
| **20mg_simvastatin (3141)** | 1 |  | 1 |  | 1 |  | 1 |  | 1 |  |
| 40mg_simvastatin (6347) | 0.99 (0.87~1.11) | 0.824 | 0.94 (0.86~1.03) | 0.199 | 1.07 (0.97~1.17) | 0.180 | 1.04 (0.92~1.17) | 0.539 | 1.01 (0.88~1.15) | 0.893 |
| 60mg_simvastatin (2) | 19216.92 (0~NA) | 0.944 | 1.58 (0.06~40.65) | 0.748 | 0.66 (0.03~16.85) | 0.768 | 3.56 (0.14~90.62) | 0.371 | 5.69 (0.22~144.69) | 0.221 |
| 10mg_atorvastatin (626) | 1.05 (0.84~1.34) | 0.664 | 0.9 (0.75~1.09) | 0.282 | 1.03 (0.86~1.25) | 0.719 | 1.21 (0.98~1.48) | 0.073 | 1.39 (1.11~1.74) | **0.004** |
| 20mg_atorvastatin (609) | 1.01 (0.8~1.28) | 0.947 | 0.93 (0.76~1.12) | 0.422 | 1.04 (0.87~1.26) | 0.658 | 1.03 (0.83~1.28) | 0.776 | 0.91 (0.7~1.17) | 0.484 |
| 5mg_rosuvastatin (121) | 0.71 (0.47~1.13) | 0.137 | 0.91 (0.61~1.33) | 0.631 | 1.05 (0.72~1.55) | 0.793 | 0.55 (0.29~0.94) | **0.042** | 0.89 (0.48~1.52) | 0.695 |
| 40mg_pravastatin (277) | 1.02 (0.73~1.44) | 0.921 | 0.67 (0.5~0.89) | **0.006** | 1.41 (1.07~1.87) | **0.016** | 1.82 (1.38~2.38) | **<0.001** | 1.81 (1.32~2.43) | **<0.001** |
| 80mg_fluvastatin (18) | 3.52 (0.71~63.8) | 0.223 | 0.42 (0.1~1.28) | 0.172 | 2.5 (0.82~10.85) | 0.150 | 3.72 (1.44~9.6) | **0.006** | 3.51 (1.28~9.02) | **0.010** |
| **AUC** | 0.5 |  | 0.515 |  | 0.517 |  | 0.507 |  | 0.501 |  |
| **Sensitivity** | **-** |  | 0.96 |  | 0.08 |  | 1 |  | 1 |  |
| **Specificity** | **-** |  | 0.07 |  | 0.95 |  | 0.02 |  | 0 |  |
| **Hosmer-Lemeshow test** | **P=0.101** |  | **P=0.752** |  | **P=0.945** |  | **P=0.142** |  | **P=0.342** |  |

Except EQD antihyperlipidemic monotherapy, we included sex, age, baseline drug-treated comorbidities, calendar year, and/or adherence in the logistic regression model.

There were no patients using 80 mg simvastatin, so we didn’t include the high level comparison.

-: No relevant data available.

**
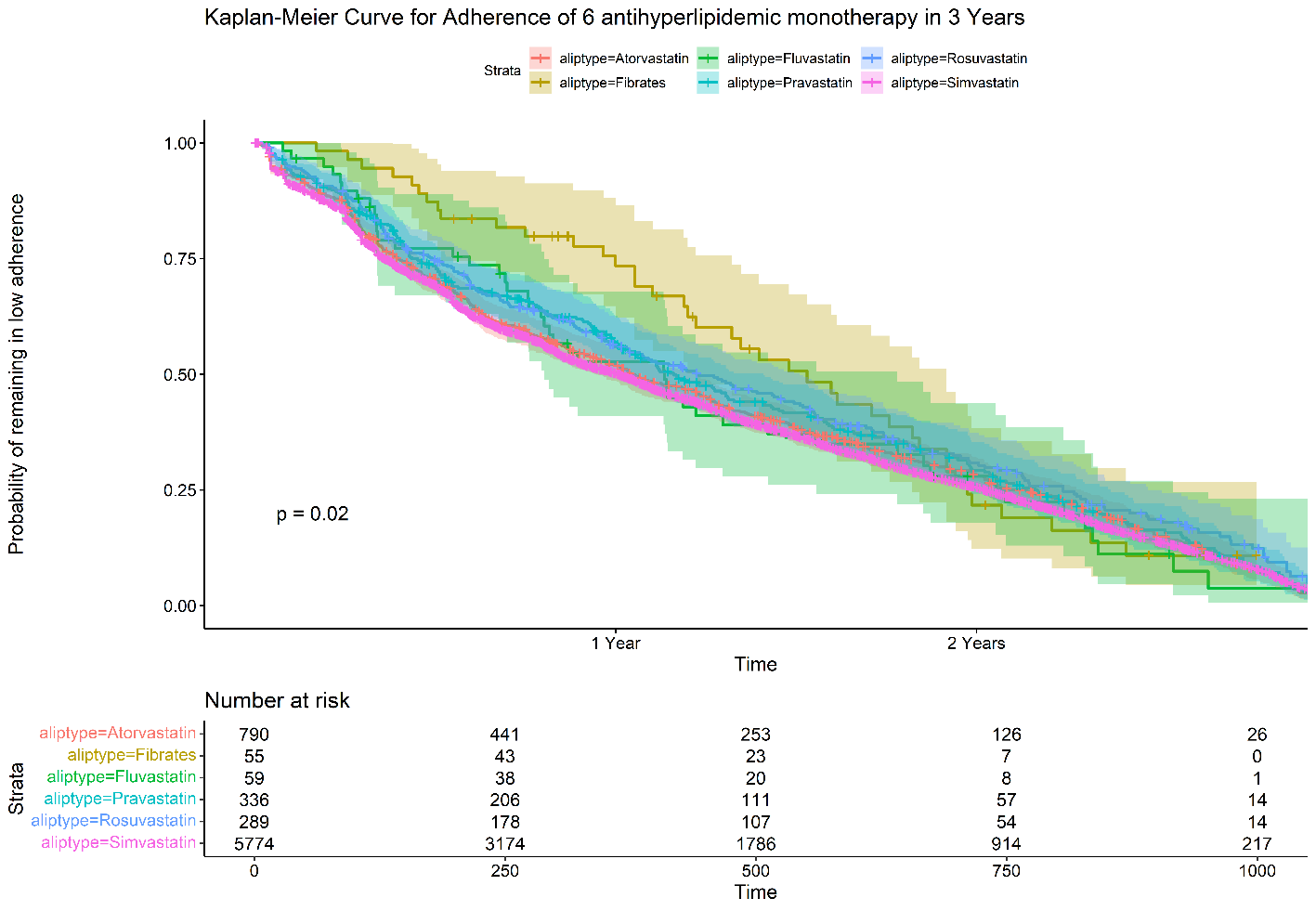
**

**a. Before IPW (low adherence as reference)**

**
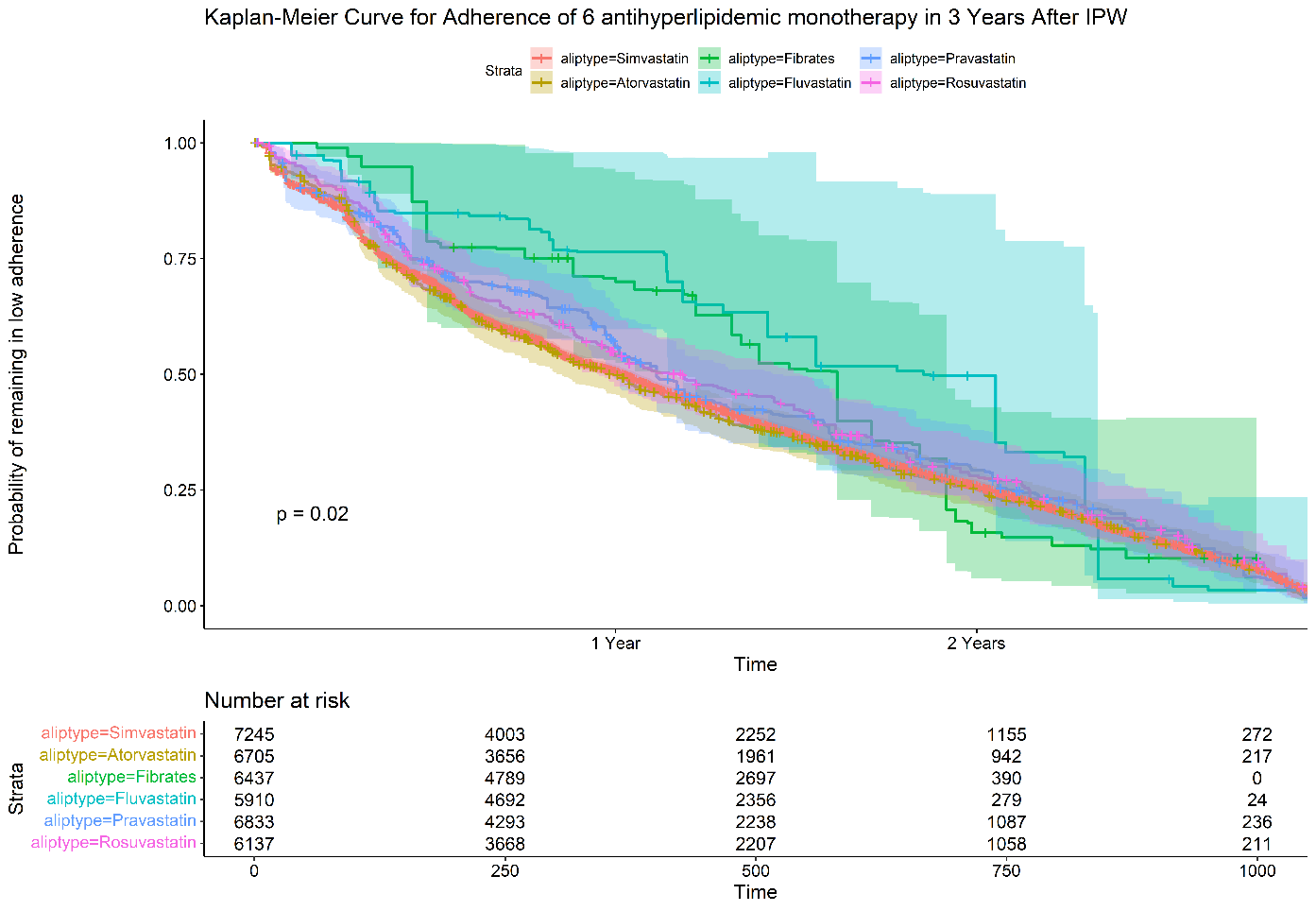
**

**b. After IPW (low adherence as reference)**

**Supplementary fig. 1** Kaplan-Meier curve for adherence of 6 classes of antihyperlipidemic drug monotherapy (7303 patients on original antihyperlipidemic monotherapy ≤3 years in 15470 patients with all drug records exceed 3 years). a. Before IPW (low adherence as reference); b. After IPW (low adherence as reference)
